# An experimental approach for real time mass spectrometric CVD gas phase investigations

**DOI:** 10.1038/s41598-017-18662-7

**Published:** 2018-01-10

**Authors:** L. Nattermann, O. Maßmeyer, E. Sterzer, V. Derpmann, H. Y. Chung, W. Stolz, K. Volz

**Affiliations:** 1 Material Sciences Center and Faculty of Physics, Philipps-Universität Marburg, Marburg, Germany; 2 Carl Zeiss SMT GmbH, Oberkochen, Germany

## Abstract

This is a report on the first setup of a recently developed, extremely sensitive and very fast 3D quadrupole ion trap mass spectrometer inline in a metalorganic vapour phase epitaxy (MOVPE) system. This setup was developed ultimately for the decomposition- and the interaction analysis of various established as well as novel metalorganic sources for MOVPE deposition of III/V semiconductors. To make *in-situ* gas phase and growth interaction analysis on a new level of sensitivity possible without disturbing the MOVPE growth process itself, an optimized experimental connection of the mass spectrometer to the MOVPE system is required. This work reports on the realization of such an experimental setup and provides first proof of concept for decomposition analysis. In addition, a comparison to previous studies and gas-phase analysis at MOVPE systems will be given in this work.

## Introduction

Metalorganic vapour phase epitaxy (MOVPE), first used for semiconductor growth on an industrial scale in the eighties^1^, is a well-established and essential method for processes in large areas of the semiconductor industry today^2,3^. From light emitting diode (LED) manufacturing and chip production to deposition of semiconductor lasers and solar cells, a multibillion dollar market is based on MOVPE^4^. Therefore, a thorough understanding of the gas phase processes as well as *in-situ* process gas analysis can help make manufacturing consistently more efficient and the development of new semiconductor materials possible. In particular, the deposition of III/V semiconductors requires high technical standards and purified metalorganic precursors (MOs), as impurities even in the parts per million (ppm) range can cause high defect densities and often lead to inoperable devices^5^. Additionally, even the smallest changes in gas phase and partial pressure ratios of the different sources can have a significant impact on the growth characteristics of semiconductor materials.

Decomposition analysis on MOs for III/V semiconductor growth was first performed in the late eighties and early nineties, when novel precursors like tertiarybutylarsine (TBAs) and triethylgallium (TEGa) entered the market. As already reported by others, these studies are highly complex^6,7^. Different experimental approaches for gas-phase investigations on MOVPE systems were tested in previous experiments. Some of the first experiments were performed by Yoshida and Watanabe^8^, who reported on mass spectrometry studies of trimethylgallium (TMGa) and TEGa decomposition reaction in H_2_ and N_2_ and introduced one of the first setups for decomposition measurements of MO sources. One experimental barrier that needs to be overcome is the different pressure regimes between the MOVPE growth system on one side and the mass spectrometer system on the other. For most studies linear quadrupole mass spectrometers (QMS) were used. Hence, at least two expensive pressure reduction stages were necessary to reduce the pressure from MOVPE conditions (mbar range) down to 1E-5 mbar needed for QMS operation^9^. Furthermore, the pressure reduction can lead to changes in the gas phase so that *in-situ* measurements become even more challenging^6^. Lee *et al*. published a study on mass spectrometry investigations on the MOVPE growth of GaAs in which they developed a setup to prevent these effects^6^. However, a disadvantage of their experimental approach is that the whole system is meant to investigate decomposition reactions and is not designed for growing semiconductors on a large scale. The challenge is that decomposition characteristics will change when growth conditions are altered^4–9^; therefore, a set-up for decomposition investigations alone is an important first step, but ultimately an in-line process analysis tool is needed to make an understanding of gas phase reactions and surface reactions under real MOVPE growth conditions possible^8,10–15^.

Additionally, previous studies showed that the addition of precursors, a change of V/III ratios, and, of course, temperature variations can lead to an alteration of the decomposition pathways and their products and therefore often to an important change of growth characteristics^13,16^. Mashita *et al*. for example, observed that the mass spectrum of a TEGa and trimethylaluminum (TMAl) mixture does not correspond to a simple sum of the spectra, measured for the two individual alkyls^9^. In addition, currently gas phase analysis of the growth of GaAs_1−x_Bi_x_ and GaP_1−x_Bi_x_ are under investigations. These MOVPE growth studies showed that the limitation of the Bi incorporation in these two different ternaries is not only explainable by strain and solubility barriers, but gas phase reactions and/or surface reactions between different precursors also seem to play a crucial role^17–19^.

In addition to the investigations on gas phase processes during the growth and the combination of established precursors, studies with novel precursors are of interest as well. Recently, several precursors have been introduced for the growth of III/V semiconductors^20–22^. Some of those precursors showed promising properties for dilute nitride III/V semiconductor growth, but they also gave rise to questions about the gas-phase interactions, as large differences compared to the growth with conventional precursors were observed. The N incorporation efficiency in GaAs for the growth with the novel N-precursor ditertiarybutylarsanoamine (DTBAA) is orders of magnitude higher than the N incorporation efficiency of the conventional N precursor unsymmetric dimethylhydrazine (UDMHy). Calculations of the relevant gas phase processes lead to the assumption that the different decomposition pathways of the respective source molecule play a significant role^20^.

Furthermore, C incorporation in III/V semiconductors is crucial in terms of device performance. As the source of C incorporation is not yet fully understood for several novel III/V semiconductors and especially for dilute nitride materials, gas phase investigations could help to understand and to decrease the C content. This becomes an even more important question because low temperature MOVPE growth has recently been gaining increased attention^23^.

In this work, we will present a new experimental setup of a real-time and highly sensitive mass spectrometer, which is directly connected to a conventional horizontal MOVPE system as the first step for future gas phase investigations of MOVPE processes. The set-up thereby enables *in-situ* MOVPE growth investigations, without disturbing or changing the growth process itself. Additionally, the set-up could have potential for CVD and ALD deposition investigations. The experimental set-up with its advantages and challenges will be discussed in detail. Furthermore, as a proof of concept, decomposition studies of tertiarybutylarsine (TBAs) will be shown and discussed in comparison to the literature^10^.

## Experimental Equipment

### Movpe

The MOVPE system used for this work is a horizontal reactor design (Aixtron Aix 200 with gas foil rotation). The system is separated into three parts, the gas supply-and mixing-cabinet, the reactor chamber with glove box for sample exchange, and the exhaust gas system. Both, H_2_ and N_2_, can be used as carrier gas and are purified to at least 9N purity. All pipes are made of electrochemical-polished stainless steel and are always kept at room temperature to prevent pre-reactions of the MOs. For group III-and group-V MOs, separate gas pipes are available for the supply of each chemical species to the reactor chamber. Hence, pre-reactions of the different MOs are minimized. The total flow rate in the reactor chamber is 6800 sccm. The graphite susceptor is located in a quartz liner. The process temperature, controlled by a thermocouple in the center of the susceptor, was calibrated by observing the phase change of an Al-coated Si-substrate. Decomposition studies were performed between room temperature and 750 °C. The reactor chamber surface area is around 720 cm^2^, including the quartz liner surface and the graphite susceptor surface (100 cm^2^).

### Mass Spectrometer

**Figure 1:**
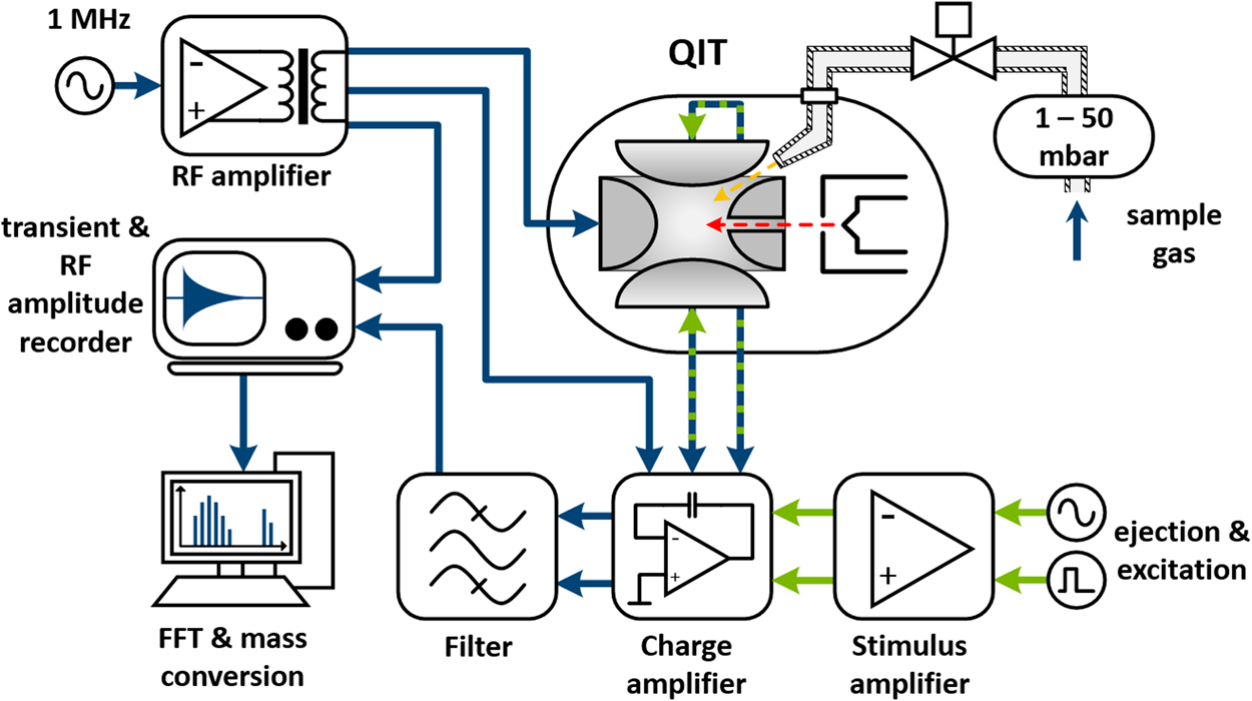
Sketch of the signal path of the quadruple ion trap based mass spectrometer. The upper right side of the figure shows the sample inlet. Gas is injected into the QIT and ionized directly inside the ion trap. An alternating electric field (upper left side) is applied to store the ions. The ions are stimulated by a short pulse and the transient of the resulting induced image current of the stimulated ions is measured (lower left part of the figure). After a Fourier transformation of this transient, the frequency of the analyzed species can be obtained and the frequencies of all species can be used to calculate the mass to charge ratios, which leads to the mass spectrum.

The instrument used here is a recently developed 3D quadruple ion trap (QIT) based mass spectrometer (Zeiss iTrap®, Carl Zeiss SMT GmbH). Figure 1 shows a sketch of the mass spectrometer and its electronics. The QIT itself is located in a stainless steel cube, which is directly connected to a turbo molecular pump that generates an ultra-high vacuum (UHV) in the measurement chamber. As an interface between the analyte source and the mass spectrometer a fast atomic layer deposition (ALD) valve, with pulse times below 50 ms, is used. Inside the QIT an integrated electron gun ionizes the analyte with an energy of 70 eV. After electron ionization (EI) the newly created ions are stored inside the QIT by applying an alternating electric field (radio frequency) to the ring electrode. Then, the stored ions are excited by a stimulus to assure a correlated movement of the ions. A highly sophisticated signal compensation concept with selective ion excitation techniques and advanced low-noise charge amplifiers close to the measurement chamber (see Fig. 1) makes it possible to detect the mirror current in the QIT induced by the oscillating ions. This mirror current is analyzed by a software tool, and after fast Fourier transformation (FFT) leads to the corresponding mass spectrum.

This mass spectrometer was chosen because it does not have any detector surfaces that can degenerate by hydrogen or covered by MO-compounds. Furthermore all surfaces were electropolished to reduce reactions with the analyte and assure reproducible measurements. This is also ensured by heating the mass analyzer to approximately 100 °C at all time. To precisely control the amount of precursor ions in the QIT, the stored waveform inverse Fourier Transform (SWIFT) technique can be applied to selectively eject ions from the QIT and to increase the sensitivity for the product ions. The resolution (Δm/m) of the instrument is better than 1000, however, due to shortcomings of the mass calibration in the current experimental setup the mass accuracy is only giving nominal masses.

## Results and Discussion

### Mass spectrometer – MOVPE setup

Firstly, the connection between the MOVPE system and mass spectrometer will be described in detail. Afterwards, a discussion of the advantages and challenges of the setup, the new mass spectrometer, and its applicability for MOVPE gas-phase investigations will follow.

**Figure 2:**
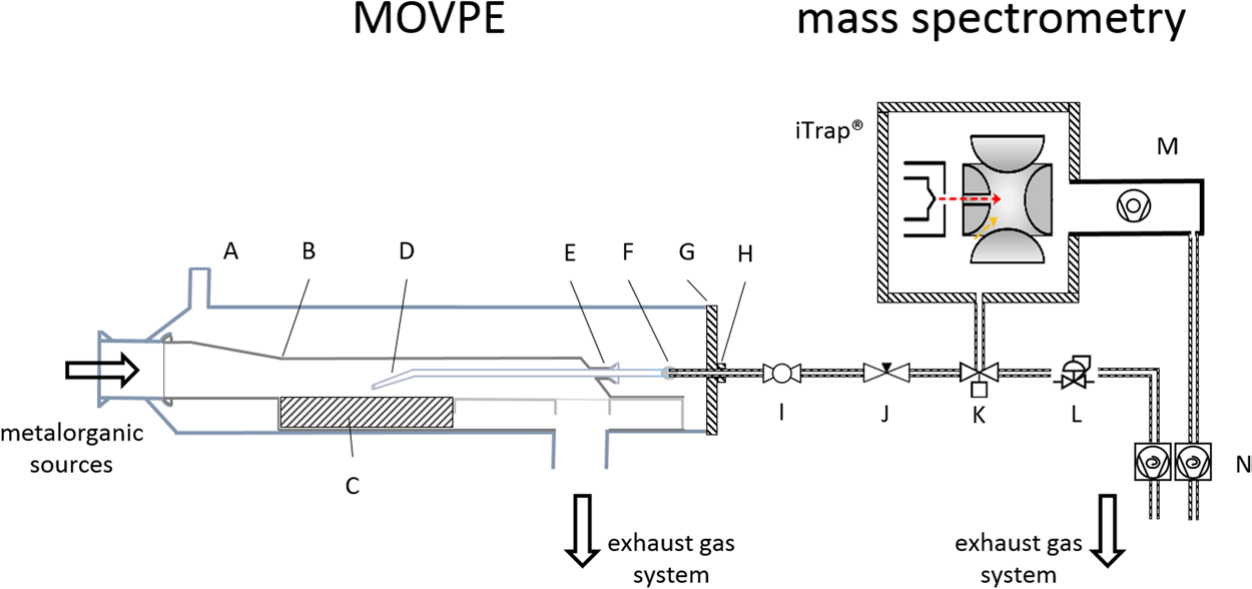
Experimental setup of MOVPE (reactor) and connection to mass spectrometer system. Liner purge (A), silica glass liner (B), susceptor (C), nozzle for analyte suction (D) quartz glass flange (E), glass-metal-transition (F), reactor gate (G), O-ring seal (H), ball valve (I), needle valve (J), ALD valve (gas supply to mass spectrometer) (K) pressure controller (L), turbo molecular pump (M), backing pumps (for analyte bypass and for turbo pump) (N).

The setup consists of three different parts (see Fig. 2): first, the adjusted MOVPE reactor and liner system; second, the bypass, which transports the analyte from the reactor chamber to the mass spectrometer and then to the exhaust gas system; and third, the mass spectrometer itself, which is also connected to the exhaust gas system. The quartz glass liner (B) in the Aixtron Aix 200 horizontal reactor (A) was extended by a glass flange (E) at the end of the liner to keep gas flow turbulences before and above the growth area as small as possible. A tapered quartz glass nozzle (D) is used to sample gas from exactly the center of the growth area, approximately 0.8 mm above the susceptor, through the extended glass flange. This nozzle is connected to a ¼ inch diameter, electrochemical-polished stainless steel pipe by a glass metal transition (F), in order to absorb the different thermal expansions of quartz glass and stainless steel. The stainless steel tube passes the reactor gate through on O-ring seal (H). This first part of the setup, located in the reactor chamber, was already successfully used by our group in previous studies^24–27^. The analyte enters the bypass. A ball valve (I) at the beginning of the bypass makes it possible to keep the bypass under constant pressure (50 mbar < bypass pressure < 5E-3 mbar) also avoiding contamination of the bypass during reactor opening and sample exchange. The ALD valve (K), which allows very short gas pulses to the mass spectrometer chamber, is located between the needle valve (J) and a pressure controller (L) to provide constant gas flow and pressure conditions for the measurements. Additionally, the bypass between the reactor gate and the pressure controller is heated to 100 °C in order to prevent condensation of the analyte on the pipe walls. After the pressure controller a backing pump (N) provides a constant gas flow through the bypass. The bypass ends at the connection of the exhaust of the backing pump to the scrubber of the MOVPE system.

The ALD valve located between needle valve and pressure controller pulses analyte into the mass spectrometer. Directly connected to the mass spectrometer, a turbo molecular pump (M) generates an ultra-high vacuum inside the measurement chamber. A second backing bump (N) (also connected to the scrubber) generates the pre-vacuum for the turbo pump.

The main challenge of gas-phase investigations of MOVPE growth processes in general is to maintain the balance between transferring the analyte as unmodified as possible into the mass spectrometer and simultaneously not influencing the MOVPE process itself. This balance has been realized in this work by operating the bypass under stable conditions, similar to the reactor pressure in the growth chamber. Expensive pressure reduction stages, which can change the analyte itself, are thereby avoided. The backing pump in combination with the needle valve and the pressure controller allows for a constant and stable flux of the analyte (between the needle valve and pressure controller) directly from the gas phase above the sample, where the decomposition takes place. Simultaneously, the flow turbulences in the reactor, caused by the nozzle, are minimized and designed to be at the very end of the growth area. This is realized by firstly inserting the nozzle at the end of the reactor chamber, and secondly by modifying the shape of the nozzle. Some turbulences cannot be avoided but are located at the very end of the growth region. The influence on the homogeneity is therefore minimal.

Additionally, the recording time for a single mass spectrum is below 2 sec, so that a high data point density and a large number of measurements for improved statistics are possible. However, experiments have shown that a recovery time of about 20 sec for the ultra-high vacuum in the mass spectrometer chamber is recommended (depending on the bypass pressure) to achieve clear spectra. Furthermore, a fine adjustment of parameters of the mass spectrometer, such as ionization time, waiting sequences, and stimulus, make additional sensitive ionization possible. Thereby, the analyte is not fractured, as for standard 70 eV EI, leading to an easier interpretation of the recorded spectra. However, the exact influence of the different parameter changes that leads to sensitive ionization conditions is not fully understood and varies for different precursors. The details of the sensitive ionization procedure for different precursors is still the subject of ongoing research and will be published elsewhere. Altogether this makes the new mass spectrometer setup in the MOVPE system a highly effective tool for investigating and controlling gas-phase processes.

In regards to the challenges, which are under examination at the moment, another important point of the described experiment is the large number of parameters of the mass spectrometer itself. Although, on the one hand, this opens up the possibility of sensitive and exact real-time measurements, on the other hand, it requires a sophisticated adjustment of all the parameters. In order to find suitable parameters for gas-phase investigations and to understand the parameter characteristics of the mass spectrometer, the well-known TBAs precursor was investigated. Results of these experiments are presented and discussed in the following as a proof-of-concept.

### Proof of concept – decomposition investigations of tertiarybutylarsine (TBAs)

#### Results

In order to verify the functionality of the experimental setup, results of TBAs investigations are presented and discussed also in comparison to literature data.

**Figure 3:**
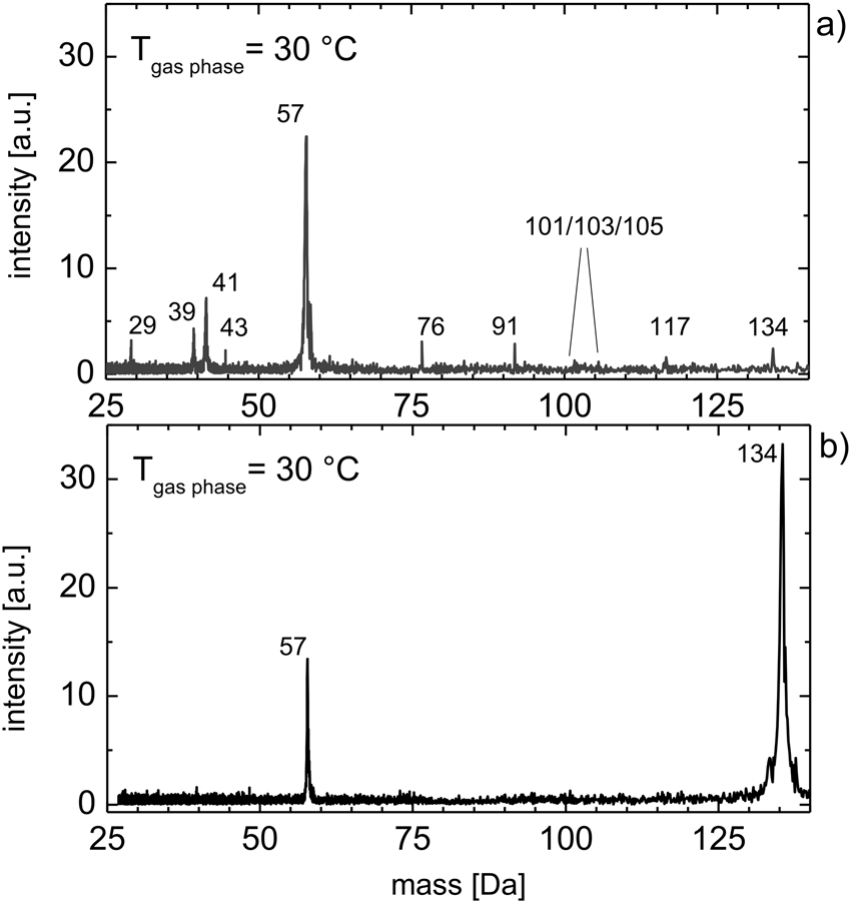
Mass spectrum of TBAs at 30 °C for standard EI conditions (**a**) and sensitive ionization (**b**). The partial pressure was 7.5E-3 mbar, the ionization potential = 70 eV.

Figure 3a shows the mass spectrum of TBAs at 30 °C with a TBAs partial pressure of 7.5E-3 mbar. Mass spectrometer parameters, such as the alternating electric field and the pulse width of the stimulus, were optimized for detection of a mass range from 15 amu to 160 amu under standard 70 eV EI conditions. The most prominent peak (principal peak) is located at 57 amu. This peak is related to ^t^Bu-radicals from the fragmentation of TBAs. But C_2_-chains (29 amu) and C_3_-chains (39, 41 and 43 amu) are also clearly visible. AsH was also detected (76 amu), as well as monomethylarsine (91 amu), dimethylarsine (101–105 amu), an additional fragmentation product at at 117 amu (loss of 2H, CH_3_) and the source molecule at 134 amu (TBAs). The spectrum in Fig. 3b was taken under sensitive ionization conditions, which means MOVPE conditions are the same as for Fig. 3a, but several mass spectrometer measurement parameters were changed to prevent strong fragmentation of the analyte. Now the parent peak of TBAs is most prominent at 134 amu. The only fragmentation product due to EI is the tBu peak at 57 amu, but also this peak is smaller than for standard 70 eV EI conditions. No further significant fragmentation products were found.

**Figure 4:**
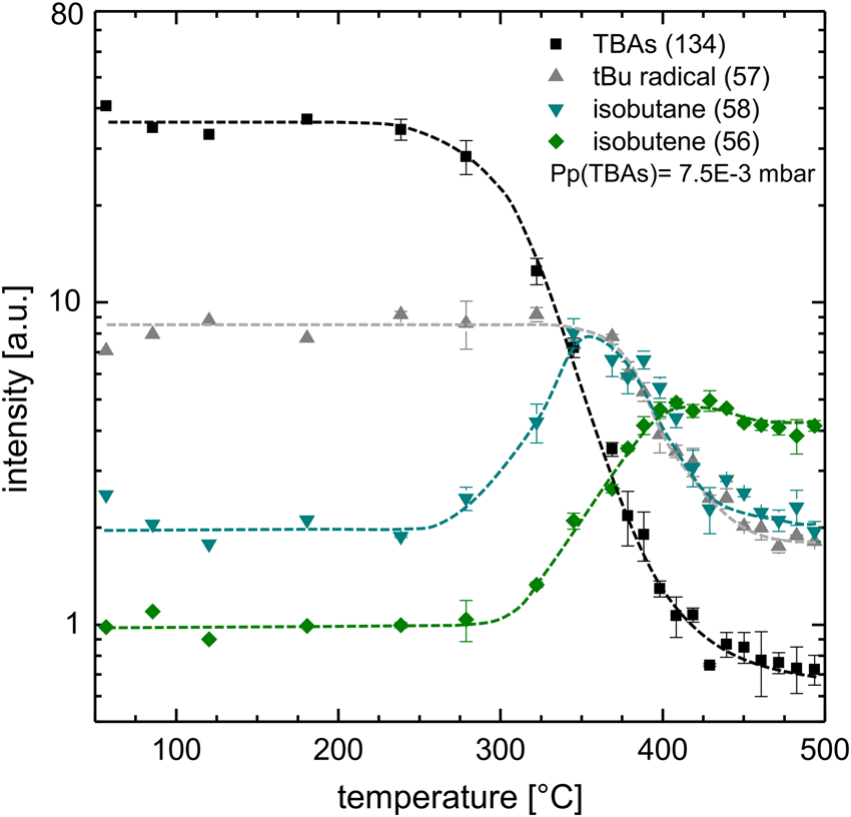
Plot of the most relevant TBAs decomposition- and fragmentation- products, the parent molecule TBAs (134 amu) the ^t^Bu-radical (57 amu) and Isobutene (56 amu) vs. temperature. The temperature was determined by correlation with data from^10^, as only the susceptor temperature could be measured during experiments.

Additionally, temperature dependent measurements of the decomposition products were performed. Results of these experiments are plotted in Fig. 4. All temperature dependent measurements are recorded without GaAs substrates or GaAs coating of the susceptor or the liner. Since the thermocouple is located in the susceptor, the gas-phase temperature was determined by the correlation of the decomposition rate with results from^6^. This temperature calibration should be valid for further decomposition and growth experiments as long as the total flux and the reactor pressure (50 mbar) are not changed. In addition, the partial pressures (range of 1E-2 mbar) of the studied precursors must be used in the very dilute limits as usually applied in MOVPE reactor systems. Three mass spectra were recorded for every temperature point from 240 °C on, where thermal decomposition begins. The intensity plotted is the mean value of these three measurements; the error bar shows the standard deviation. The most relevant decomposition products of TBAs are isobutene (C_4_H_8_) and isobutane (C_4_H_10_)^28^. The intensity could be monitored by mass 56 amu and 58 amu directly, since sensitive ionization conditions were used, which is different from previous studies in which isobutane was tracked by 43 amu^10,29^. At around 250 °C gas-phase temperature, the TBAs signal decreases with increasing temperature. The parent peak disappears fully at around 450 °C. Increasing isobutane production starts slightly below 300 °C, and the isobutene signal starts to increase at 325 °C, about 50 °C higher as compared to the isobutane signal. The isobutane and isobutene intensity below 250 °C is at noise level. The ^t^Bu radical (57 amu) decreases at slightly higher temperatures of 350 °C. Its temperature dependence is following the isobutane signal and decreases down to a constant level at 450 °C.

#### Comparison to literature

The influence of the EI process on the analyte is always important and the first step that needs to be taken into account in order to interpret the mass spectra. Various databases of fragmentation spectra of different molecules and fragments are available for the 70 eV EI technique. For these studies the database *Chemistry WebBook* of the *National Institute of Standards and Technology (NIST)* was used for identification^29^. The results observed are in agreement with the literature^10,30,31^. In the study published by Larsen, Buchan and Stringfellow^6^, the same fragmentation spectrum was found for TBAs at room temperature. This is an important finding, as it shows that the results obtained with the experimental setup discussed here, are suitable for direct comparison with data from previous studies. It also indicates that established mass spectrometer databases can be used for interpretation of mass spectra collected with the new mass spectrometer, an understanding that was not clear in the first place, since different ionization techniques often lead to a different fragmentation of molecules during the ionization process in mass spectrometers (see 2.2). This enables a straightforward comparison to previous studies. However, the fragmentation of the analyte due to EI often makes interpretation of the obtained mass spectra more complicated, especially when temperature dependent measurements are performed and the thermal decomposition of the analyte is under investigation. Figure 3b shows a spectrum of TBAs, also recorded at 30 °C as in Fig. 3a, but under fine adjustment of mass spectrometer parameters for sensitive ionization conditions. One can see that the only fragmentation product is the ^t^Bu radical (57 amu) and that the most prominent peak comes from the TBAs (134 amu) itself. Thereby, it is possible to directly interpret the mass spectra by the weight of the molecules produced due to thermal decomposition.

The results of the temperature dependent measurements in Fig. 4 provide insights into the thermal decomposition characteristics of TBAs. While isobutane and isobutene are products of the thermal decomposition of the TBAs molecule, the ^t^Bu radicals are both a fragmentation product of the EI and a decomposition product of the thermal induced decomposition of the precursor at higher temperatures. In previous studies TBAs was tracked by the 57 amu peak as the parent molecule was mostly fractured by EI^10^. The sensitive ionization used here makes it possible to distinguish between TBAs and ^t^Bu molecules as can be seen in Fig. 4. From room temperature up to 250 °C, the concentration of the all products is caused by a small number of fractured TBAs molecules. From 250 °C, the spectra change when the thermal decomposition of the TBAs starts. The ^t^Bu concentration (57 amu) decreases slightly after the TBAs due to the thermal induced generation of free radicals. The fact that the isobutane signal (58 amu) tracks the ^t^Bu signal from about 375 °C could also be caused by EI cracking of an H atom from the isobutane molecules.

The observation that the production of isobutane starts slightly before the generation of isobutene confirms the findings of Larsen *et al*. and Lee *et al*.^10,28^. They found that the abundance of isobutane dominates at lower temperatures before the production of isobutene as a product of β-H-elimination increases significantly at temperatures above 400 °C. Also Foster *et al*. and Zimmermann *et al*. reported that there are two decomposition pathways, first a free radical process producing isobutane and second a β-H-elimination process, that generates isobutene as a side product^30,31^. Hence, the observations made in these experiments as well confirm β-H-elimination as the main decomposition mechanism at temperatures above 390 °C^32,33^.

All findings agree well with previous experimental investigations, so that the functionality of the setup discussed here is confirmed to be suitable for future gas phase and decomposition investigations also of more complex and novel precursor molecules. Nevertheless, one has to consider that there can occur slight changes of the analyte species on their way from the reactor chamber into the mass spectrometer. Although the bypass between reactor chamber and mass spectrometer was heated to 100 °C, gas flux velocities were high and kept constant, and the ionization of the analyte happened right after entering the mass spectrometer on a millisecond range, slight changes of the analyte could not be completely avoided. However, ultimately the TBAs measurements and a positive comparison to literature data, which include the evidence of highly reactive species, show that this setup is suitable to detect and analyze decomposition products, and can enable deeper insights into the behavior of metal organic precursors during MOCVD.

## Summary

A new setup for inline gas-phase investigations on a MOVPE system was introduced. The characteristics, advantages, and challenges of the developed experimental setup were illustrated and discussed in context of previous experimental studies on metalorganic decomposition pathways. TBAs experiments showed good agreement with the literature. Furthermore, it was possible to demonstrate sensitive ionization conditions which make direct interpretation of the data possible, as the ionization fragmentation of the analyte is minimized. Altogether, that makes the set-up discussed here a promising tool for future MOVPE gas phase processes, and could have an application potential also for CVD and ALD *in-situ* deposition investigations.
